# Clinicopathological factors associated with HER2 status in gastric cancer: results from a prospective multicenter observational cohort study in a Japanese population (JFMC44-1101)

**DOI:** 10.1007/s10120-015-0518-8

**Published:** 2015-08-12

**Authors:** Satoshi Matsusaka, Atsushi Nashimoto, Kazuhiro Nishikawa, Akira Miki, Hiroto Miwa, Kazuya Yamaguchi, Takaki Yoshikawa, Atsushi Ochiai, Satoshi Morita, Takeshi Sano, Yasuhiro Kodera, Yoshihiro Kakeji, Junichi Sakamoto, Shigetoyo Saji, Kazuhiro Yoshida

**Affiliations:** Cancer Institute Hospital of the Japanese Foundation for Cancer Research, Tokyo, Japan; Department of Surgery, Niigata Prefectural Cancer Center Niigata Hospital, Niigata, Japan; Department of Surgery, Osaka General Medical Center, Osaka, Japan; Department of Surgery, Kobe City Medical Center General Hospital, Kobe, Japan; Division of Gastroenterology, Department of Internal Medicine, Hyogo College of Medicine, Nishinomiya, Japan; Department of Surgical Oncology, Gifu University Graduate School of Medicine, 1-1 Yanagido, Gifu, 501-1194 Japan; Department of Gastrointestinal Surgery, Kanagawa Cancer Center, Yokohama, Japan; Research Center for Innovative Oncology, National Cancer Center Hospital East, Chiba, Japan; Kyoto University Graduate School of Medicine, Kyoto, Japan; Department of Gastroenterological Surgery (Surgery II), Nagoya University Graduate School of Medicine, Nagoya, Japan; Division of Gastrointestinal Surgery, Department of Surgery, Graduate School of Medicine, Kobe University, Kobe, Japan; Japanese Foundation for Multidisciplinary Treatment of Cancer, Tokyo, Japan

**Keywords:** Fluorescence in situ hybridization, Stomach neoplasms, Human *ERBB2* protein, Immunohistochemistry

## Abstract

**Background:**

Human epidermal growth factor (HER) 2 positivity and its association with clinicopathological factors remain unclear in Japanese gastric cancer (GC) patients. We performed a prospective, multicenter, observational cohort study to evaluate HER2 protein expression and gene amplification in Japanese metastatic and recurrent GC patients, and explored its correlations with clinicopathological features.

**Methods:**

HER2 protein expression and gene amplification were centrally assessed in formalin-fixed, paraffin-embedded GC tissue by immunohistochemistry (IHC) and fluorescence in situ hybridization (FISH). Patient information was collected, and associations between clinicopathological factors and HER2 positivity (IHC score 3+ and/or FISH positive) and low HER2 expression (IHC score 0/FISH positive or IHC score 1+/FISH positive) were examined.

**Results:**

From September 2011 to June 2012, 1461 patients were registered across 157 sites, and the HER2 status of 1427 patients was evaluated. The rate of HER2 positivity was 21.2 %, whereas the rate of high HER2 expression (IHC score 2+/FISH positive or IHC score 3+) was 15.6 % and that of low HER2 expression was 7.0 %. Multiple logistic regression analysis identified intestinal type, absence of peritoneal metastasis, and hepatic metastasis as significant independent factors related to HER2 positivity. The intestinal type was confirmed to be the GC subtype predominantly associated with lower HER2 expression. Sampling conditions including number of biopsy samples, formalin concentration, and formalin-fixation time did not significantly affect HER2 positivity.

**Conclusions:**

HER2 expression in Japanese patients was comparable to that in other populations examined. Intestinal type was an independent factor related to HER2 positivity and low HER2 expression.

## Introduction

Trastuzumab (Herceptin) is a monoclonal antibody that specifically targets human epidermal growth factor receptor 2 (HER2), a receptor associated with gastric cancer (GC) tumorigenesis, by directly binding its extracellular domain [1]. The Trastuzumab for GAstric Cancer (ToGA) study, an open-label, international, multicenter, phase III, randomized controlled trial, examined the clinical efficacy and safety of trastuzumab combined with standard chemotherapy (capecitabine or intravenously administered 5-fluorouracil and cisplatin) for first-line treatment of HER2-overexpressing advanced gastric or gastroesophageal junction cancers. Addition of trastuzumab therapy to chemotherapy improved median survival (13.8 months) compared with chemotherapy alone (11.1 months) (*P* = 0.0046), and showed significant improvements in time to progression and progression-free survival in the trastuzumab-treated group, with a comparable toxicity profile [2]. As a result, trastuzumab therapy plus chemotherapy has become the standard treatment for HER2-positive advanced GC patients, as determined by immunohistochemistry (IHC) and/or fluorescence in situ hybridization (FISH). In Japan and the USA, trastuzumab is approved for patients with metastatic GC whose tumors are HER2 positive, as defined by a positive FISH result or an IHC score of 3+. In the European Union, however, trastuzumab is recommended only for individuals whose tumors have high HER2 protein expression, as defined by an IHC score of 2+/positive FISH result or an IHC score of 3+ based on the subset analysis of the ToGA study. HER2 evaluation has therefore become an important approach for predicting clinical efficacy of trastuzumab. The variation in the HER2-positivity rate between countries possibly reflects the unstandardized testing modality and other country-specific factors; it was identified as 27 % in Japanese patients in the ToGA study [3, 4], which was higher than that identified in previous studies in Japan [5–7].

In the ToGA study, the strong effect of trastuzumab was evident in patients with higher HER2 protein expression (IHC score 2+/FISH positive or IHC score 3+), whereas the efficacy was unclear in patients with low HER2 expression (IHC score 0/FISH positive or IHC score 1+/FISH positive). These results were obtained via a subgroup analysis, and may be affected by the smaller number of patients with low HER2 expression than higher HER2 protein expression. Thus, it is premature to conclude that addition of trastuzumab therapy to chemotherapy is not beneficial in patients with low HER2 expression. Additionally, little has been reported about the clinicopathological features of patients with low HER2 expression [8–10].

In unresectable cases, tumor behavior before treatment is evaluated by biopsy specimens. However, because GC is considered a mixture of heterogeneous tumor types, small biopsy specimens may not reflect its overall behavior, and few studies have focused on HER2-positivity concordance between diagnostic biopsy specimens and surgical specimens [11, 12]. Because of tumor heterogeneity, the accuracy of HER2 testing can be affected by the site of the examined HER2-stained cells; thus, gastric biopsies could yield false-negative results [13].

We performed a prospective, multicenter, observational cohort study (JFMC44-1101) to evaluate HER2 expression and gene amplification in consecutively registered Japanese patients with metastatic (excluding curatively resected cases) or recurrent GC, and explored the clinicopathological features in relation to HER2 positivity (IHC score 3+ and/or FISH positive) or low HER2 expression (IHC score 0/FISH positive or IHC score 1+/FISH positive). Furthermore, we evaluated the relationship between HER2 protein expression/gene amplification and sampling conditions to ascertain whether HER2 positivity in GC patients can be accurately determined from routinely prepared formalin-fixed, paraffin-embedded tissues.

## Methods

### Patients

JFMC44-1101 is a multicenter, observational cohort study to evaluate HER2 protein expression and gene amplification in consecutively registered Japanese patients with metastatic (excluding curatively resected cases) or recurrent GC. This trial was approved by the central ethics committee of the Japanese Foundation for Multidisciplinary Treatment of Cancer (JFMC) and the institutional review boards of all participating centers. In total, 1427 cases of GC were studied, of which 396 cases were proximal and 1031 were distal. Patients were classified into two groups on the basis of age (younger than 65 years or 65 years or older), according to the WHO classification [14]. All patients provided written informed consent before undergoing study-specific screening procedures. The trial was registered with the University Hospital Medical Information Network (UMIN) Clinical Trials Registry (UMIN ID UMIN000006190). Patient information was collected on the basis of the Japanese Classification of Gastric Carcinoma (third English edition) [15].

### Selection criteria

Eligible patients were aged 20 years or older with histologically confirmed adenocarcinoma and in whom metastatic or recurrent GC had been diagnosed after August 2011. Additional eligibility criteria included available pathological tissue samples (six 4-μm-thick tissue sections), and written informed patient consent and consent to disseminate the clinical data.

### HER2 evaluation

Excised tissue was formalin fixed and paraffin embedded by conventional histological methods. Six 3–5-µm sections were submitted per paraffin-embedded tissue block to allow assessment of the HER2 status: one section was used for each of hematoxylin and eosin staining, IHC, IHC negative control, and FISH, and the remaining two sections were retained as backup specimens. HER2 evaluation was performed centrally with an in vitro diagnosis kit validated by the Japanese Ministry of Health, Labour and Welfare, according to the manufacturer’s procedure as follows: tissue sections were tested for HER2 status by IHC with the PATHWAY anti-HER2 (4B5) rabbit monoclonal primary antibody (Roche Diagnostics, Tokyo, Japan), and by FISH with a PathVysion HER-2 DNA probe kit (Abbott Japan, Tokyo, Japan). IHC and FISH results were interpreted centrally, and HER2 positivity was defined as an IHC score of 3+ and/or a positive FISH result in accordance with the ToGA study parameters [2]. High HER2 expression was defined as an IHC score of 2+/positive FISH result or an IHC score of 3+, and low HER2 expression was defined as an IHC score of 0/positive FISH result or an IHC score of 1+/positive FISH result. The IHC scoring criteria were as follows: IHC score 0, no staining or membrane staining in less than 10 % of invasive tumor cells; IHC score 1+, weak membrane staining in 10 % or more of invasive tumor cells; IHC score 2+, weak to moderate complete or basolateral membrane staining in 10 % or more of invasive tumor cells; and IHC score 3+, moderate to strong complete or basolateral membrane staining in 10 % or more of invasive tumor cells. To determine FISH-positive status, we determined the fluorescence signal ratio of HER2 (orange) to chromosome enumeration probe 17 (CEP17; green) by counting 20 cancer cells under a fluorescence microscope with a ×100 objective lens. A sample was considered negative for gene amplification (FISH negative) if the HER2-to-CEP17 ratio was less than 2.0, and positive for gene amplification (FISH positive) if the ratio was 2.0 or greater. A HER2-to-CEP17 ratio of 1.8–2.2 (inclusive) was considered equivocal, and was found in 40 cancer cells. Samples were evaluated with a conventional histopathology method, and associations between clinicopathological factors and HER2 positivity or low HER2 expression were examined.

### Statistical analysis

Data were analyzed with the Statistical Package for SAS version 9.2 (SAS Institute, Cary, NC, USA). Fisher’s test, Wilcoxon’s test, and the chi-squared test were used to test the association between HER2 status and clinicopathological characteristics. To assess the association of HER2 status with clinicopathological features, univariate and multivariate logistic regression analyses were performed. Confidence intervals were computed with the normal approximation of the binomial distribution.

## Results

### Patient and sample characteristics

**Fig. 1:**
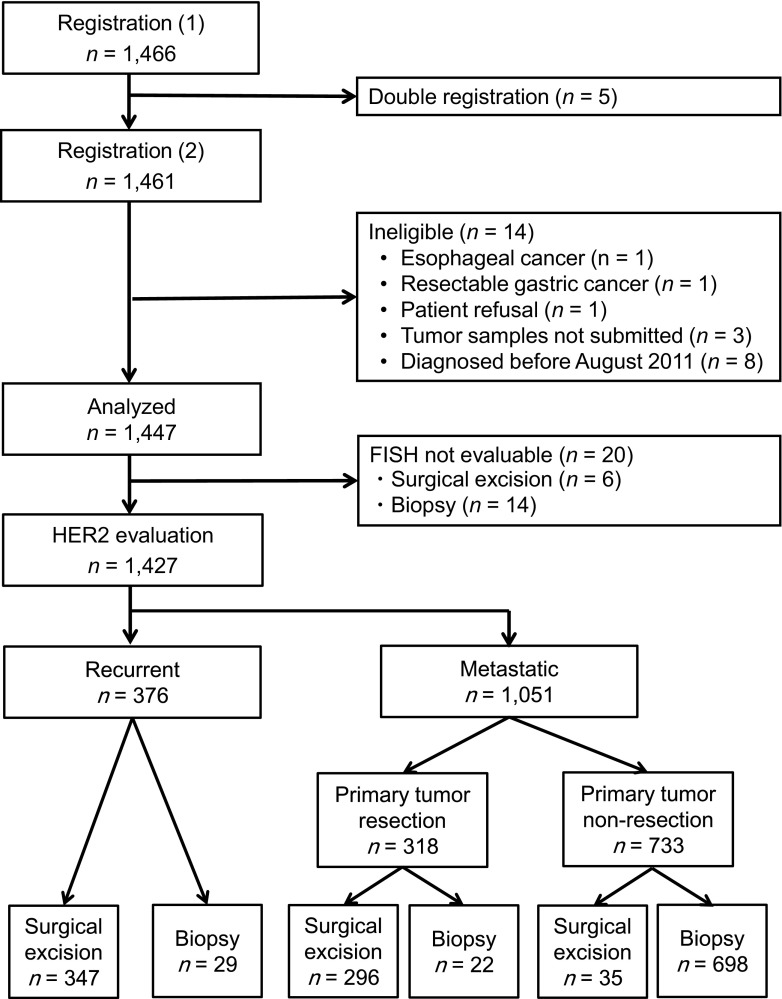
Trial profile. Human epidermal growth factor receptor 2 (*HER2*) evaluation by immunohistochemistry and fluorescence in situ hybridization (*FISH*) in 1427 samples

**Fig. 2:**
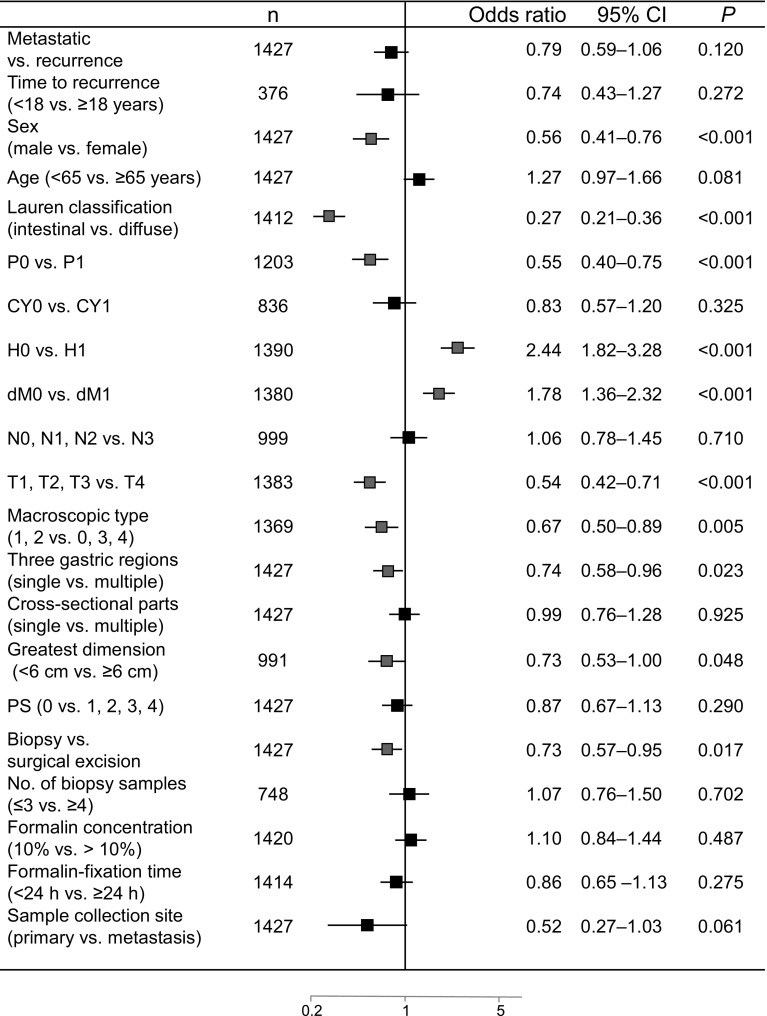

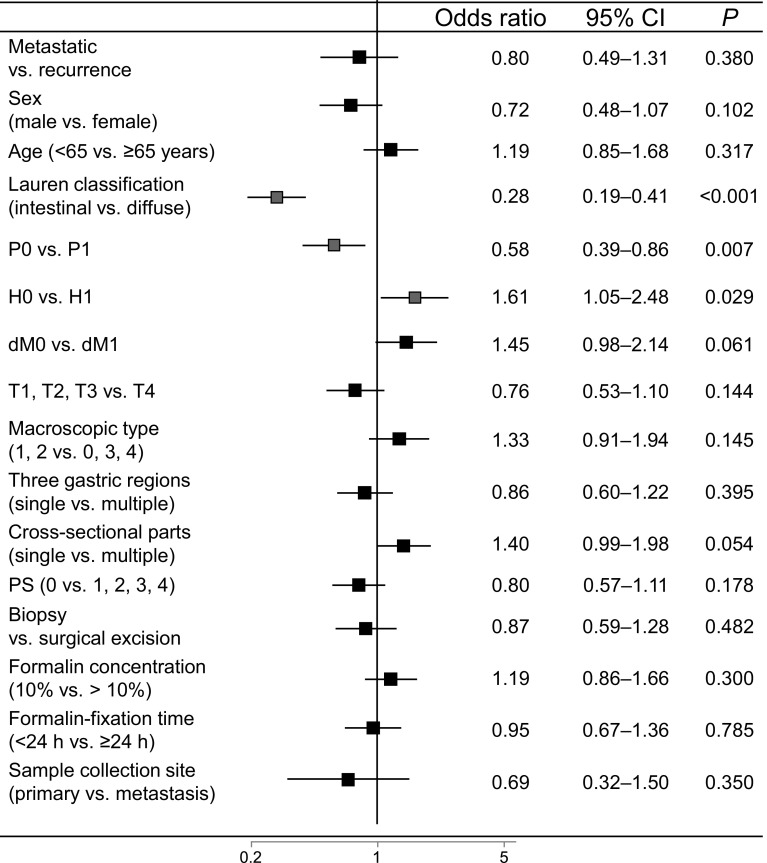
Correlation of human epidermal growth factor receptor 2 (HER2) positivity with clinicopathological factors. **a** Univariate analysis of HER2 positivity (immunohistochemistry score 3+ and/or fluorescence in situ hybridization positive) in samples from gastric cancer (GC) patients. **b** Multivariate analysis of HER2-positivity in samples from GC patients (*n* = 1088). *Red squares* indicate a significant association with HER2 status (HER2 positive/negative). All *P* values are two-sided, with *P* < 0.05 indicating statistical significance. *CI* confidence interval, *CY* peritoneal lavage cytology, *dM* distant metastasis excluding that detected in the peritoneum, by peritoneal lavage cytology, and in the liver, *H* hepatic metastasis, *N* lymph node metastasis, *P* peritoneal metastasis, *PS* performance status, *T* depth of tumor invasion (color figure online)

The trial profile is summarized in Fig. 1. A total of 1461 patients from 157 sites were registered between September 2011 and June 2012. Of these, the HER2 status of 1427 patients was evaluated by both IHC and FISH. Samples were collected from the major tumor site in each patient and were categorized as proximal if they were located in the upper third of the stomach or in the esophagus, and distal if they were situated in the middle third or lower third of the stomach; 27.8 % (396/1427) were proximal GCs and 72.2 % (1031/1427) were distal GCs. Patient and sample characteristics at the baseline are summarized in Table 1. The median age of the patients was 68 years (range 23–99 years). The correlations between patient or sample characteristics and HER2 status are summarized in Table 2. Histopathological groupings based on the Lauren classification revealed that 642 patients had intestinal-type tumors and 770 had diffuse-type tumors. Samples were obtained via surgical excision (678 patients) or biopsy (749 patients), and sample collection sites consisted of primary tumors (1348 patients) or metastatic regions (79 patients). HER2-positivity rates in surgically resected tumors and biopsy specimens were significantly different at 18.4 and 23.6 % (Fisher’s test, *P* = 0.016), respectively (Table 2). In univariate analysis, the factor biopsy specimen was found to be significantly associated with HER2 positivity (Fig. 2a). However, this association was lost in the multivariate analysis (Fig. 2b).

**Table 1:** Characteristics of gastric cancer (GC) patients (*n* = 1427)

	Recurrent GC	Metastatic GC	
		Primary tumor resection	Primary tumor no resection
	*n* = 376	*n* = 318	*n* = 733
Sex			
Male	276 (73.4 %)	215 (67.6 %)	529 (72.2)
Female	100 (26.6 %)	103 (32.4 %)	204 (27.8)
Age			
Median (years)	68		
Range (years)	23–99		
<65 years	158 (42.0 %)	108 (34.0 %)	273 (37.2 %)
≥65 years	218 (58.0 %)	210 (66.0 %)	460 (62.8 %)
PS (ECOG)			
0	242 (64.4 %)	180 (56.6 %)	438 (59.8 %)
1, 2, 3, 4	134 (35.6 %)	138 (43.4 %)	295 (40.2 %)
Source of sample			
Biopsy	29 (7.7 %)	22 (6.9 %)	698 (95.2 %)
Surgical excision	347 (92.3 %)	296 (93.1 %)	35 (4.8 %)
Depth of tumor invasion			
T1a	4 (1.1 %)	0	1 (0.1 %)
T1b	19 (5.1 %)	4 (1.3 %)	4 (0.5 %)
T2	45 (12.0 %)	11 (3.5 %)	20 (2.7 %)
T3	121 (32.2 %)	36 (11.3 %)	142 (19.4 %)
T4a	153 (40.7 %)	200 (62.9 %)	367 (50.1 %)
T4b	33 (8.8 %)	63 (19.8 %)	160 (21.8 %)
Tx	0	4 (1.3 %)	39 (5.3 %)
Unclear	1 (0.3 %)		
Lymph node metastasis			
N0	65 (17.3 %)	22 (6.9 %)	93 (12.7 %)
N1	57 (15.2 %)	39 (12.3 %)	44 (6.0 %)
N2	76 (20.2 %)	43 (13.5 %)	108 (14.7 %)
N3a	106 (28.2 %)	75 (23.6 %)	83 (11.3 %)
N3b	61 (16.2 %)	102 (32.1 %)	25 (3.4 %)
NX	11 (2.9 %)	37 (11.6 %)	380 (51.8 %)
Peritoneal metastasis			
P0	358 (95.2 %)	159 (50.0 %)	192 (26.2 %)
P1	10 (2.7 %)	151 (47.5 %)	333 (45.4 %)
Unclear	8 (2.1 %)	8 (2.5)	208 (28.4 %)
Peritoneal lavage cytology			
CY0	316 (84.0 %)	115 (36.2 %)	85 (11.6 %)
CY1	0	159 (50.0 %)	161 (22.0 %)
Unclear	60 (16.0 %)	44 (13.8 %)	487 (66.4 %)
Hepatic metastasis			
H0	371 (98.7 %)	257 (80.8 %)	493 (67.3 %)
H1	3 (0.8 %)	57 (17.9 %)	209 (28.5 %)
Unclear	2 (0.5 %)	4 (1.3 %)	31 (4.2 %)

*ECOG* Eastern Cooperative Oncology Group, *PS* performance status

**Table 2:** Correlation between patient and sample characteristics and human epidermal growth factor receptor 2 (*HER2*) status (*n* = 1427)

	Number	HER2 positivity (%)	HER2 positive (*n* = 302)	HER2 negative (*n* = 1125)
Diagnosis status				
Metastatic	1051	22.2	233	818
Recurrent	376	18.4	69	307
Time to recurrence				
<18 months	212	20.3	43	169
≥18 months	164	15.9	26	138
Sex				
Male	1020	23.7	242	778
Female	407	14.7	60	347
Age				
<65 years	539	18.7	101	438
≥65 years	888	22.6	201	687
Tumor location: three gastric regions (major site)				
U	391	21.2	83	308
M	548	19.9	109	439
L	480	22.3	107	373
Other (E or D)	6	33.3	2	4
Tumor location: cross-sectional part (major site)				
Less	550	22.0	121	429
Gre	202	23.8	48	154
Ant	142	21.8	31	111
Post	188	20.2	38	150
Circ	332	18.1	60	272
Macroscopic type				
Type 0	46	28.3	13	33
Type 1	40	30.0	12	28
Type 2	291	26.5	77	214
Type 3	639	23.6	151	488
Type 4	353	11.3	40	313
Type 5	55	14.5	8	47
Histological classification^a^				
pap	38	36.8	14	24
tub1	155	38.1	59	96
tub2	353	33.1	117	236
por1	359	19.8	71	288
por2	347	7.8	27	320
sig	134	6.7	9	125
muc	41	12.2	5	36
Lauren classification^b^				
Intestinal	642	32.7	210	432
Diffuse	770	11.7	90	680
Peritoneal metastasis				
P0	709	23.1	164	545
P1	494	14.2	70	424
Peritoneal lavage cytology				
CY0	516	18.6	96	420
CY1	320	15.9	51	269
Hepatic metastasis				
H0	1121	18.0	202	919
H1	269	34.9	94	175
Distant metastasis^c^				
dM0	934	18.0	168	766
dM1	446	28.0	125	321
Lymph node metastasis				
N0	180	12.8	23	157
N1	140	23.6	33	107
N2	227	21.6	49	178
N3a	264	24.6	65	199
N3b	188	13.8	26	162
Depth of tumor invasion				
T1	32	37.5	12	20
T2	76	25.0	19	57
T3	299	28.4	85	214
T4a	720	16.5	119	601
T4b	256	21.5	55	201
Source of sample				
Surgical excision	678	18.4	125	553
Biopsy	749	23.6	177	572
No. of biopsy samples				
1–3	339	23.0	78	261
4–8	378	24.1	91	287
≥9	31	25.8	8	23
Formalin concentration				
10 %	950	20.7	197	753
15 %	129	16.3	21	108
20 %	335	25.1	84	251
>20 %	6	0.0	0	6
Formalin fixation time				
<18 h	497	22.9	114	383
≥18 h, <24 h	462	21.2	98	364
≥24 h, <48 h	269	17.5	47	222
≥48 h	186	22.6	42	144
Sample collection sites				
Primary tumor	1348	21.7	292	1056
Metastatic region	79	12.7	10	69

*Ant* anterior wall, *Circ* circumferential, *D* duodenum, *E* esophagus, *Gre* greater curvature, *L* lower third, *Less* lesser curvature, *M* middle third, *muc* mucinous adenocarcinoma, *pap* papillary adenocarcinoma, *por1* solid-type poorly differentiated adenocarcinoma, *por2* non-solid-type poorly differentiated adenocarcinoma, *Post* posterior wall, *U* upper third, *sig* signet ring cell carcinoma, *tub1* well-differentiated tubular adenocarcinoma, *tub2* moderately differentiated tubular adenocarcinoma

^a^Histological features were classified on the basis of the Japanese Classification of Gastric Carcinoma (third English edition)

^b^For Lauren classification, pap, tub, and por1 of type 1 or type 2 were defined as intestinal type, and the others were defined as diffuse type

^c^Distant metastasis was defined as metastasis to other organs excluding that detected in the peritoneum, by peritoneal lavage cytology, and in the liver

### HER2 positivity and correlation with clinicopathological factors

The overall HER2-positivity rate (IHC score 3+ and/or FISH positive) was 21.2 % [95 % confidence interval (CI) 19.1–23.4; 302 of 1427 patients]. There was no significant difference (*P* = 0.885; Fisher’s exact test, two-sided) in HER2 positivity between proximal GC cases (21.5 %; 85 of 396 cases) and distal GC cases (21.0 %; 217 of 1031 cases). The incidence of high HER2 protein expression (IHC score 3+ or IHC score 2+ and FISH positive) was 15.6 % (223 of 1427 patients). FISH was positive in 47.3 % of IHC score 2+ cases (61 of 129 patients) and 97.5 % of IHC score 3+ cases (158 of 162 patients) (Table 3). In the univariate analysis, HER2 positivity was significantly correlated with sex, histological type, peritoneal metastasis, hepatic metastasis, distant metastasis excluding that detected in the peritoneum, by peritoneal lavage cytology, and in the liver, depth of tumor invasion, macroscopic type, primary tumor location, size, and sample source (Fig. 2a). Multivariate logistic regression analysis revealed that intestinal type, absence of peritoneal metastasis, and hepatic metastasis were independent factors related to HER2 positivity (Fig. 2b). Sampling conditions such as number of biopsy samples, formalin concentration, formalin-fixation time, and sample source had no significant effect on HER2 positivity.

**Table 3:** Human epidermal growth factor receptor 2 positive rates as assessed by fluorescence in situ hybridization (*FISH*) and immunohistochemistry (*IHC*)

FISH result	IHC score				Total
	0	1+	2+	3+	
Negative	573 (96.8 %)	484 (89.0 %)	68 (52.7 %)	4 (2.5 %)	1129 (79.1 %)
Positive	19 (3.2 %)	60 (11.0 %)	61 (47.3 %)	158 (97.5 %)	298 (20.9 %)
Total	592 (100 %)	544 (100 %)	129 (100 %)	162 (100 %)	1427 (100 %)

### Correlation of HER2 gene amplification by FISH with clinicopathological factors in IHC score 0/1+ cases

**Fig. 3:**
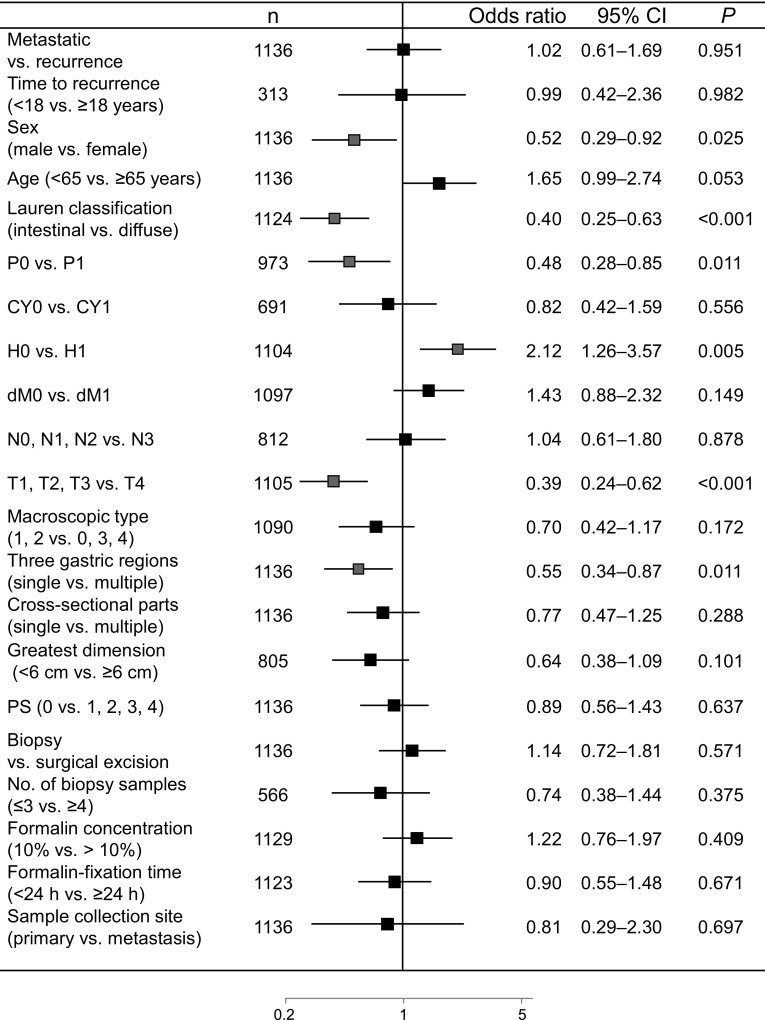

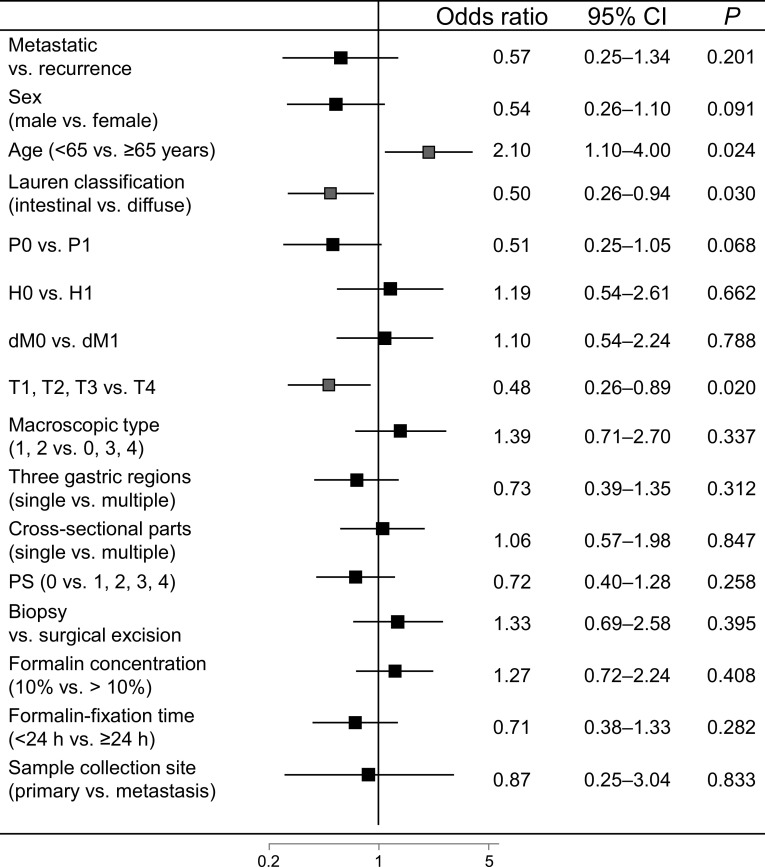
Correlation of human epidermal growth factor receptor 2 (HER2) gene amplification assessed by fluorescence in situ hybridization (FISH) with clinicopathological factors in immunohistochemistry (IHC) score 0/1+ cases. **a** Univariate analysis of low HER2 expression as assessed by IHC score 0/FISH-positive or IHC score 1+/FISH-positive samples from gastric cancer patients. **b** Multivariate analysis of low HER2 expression as assessed by IHC score 0/FISH-positive or IHC score 1+/FISH-positive samples from gastric cancer patients (*n* = 874). *Red squares* indicate a significant association with HER2 status (IHC score 0/FISH positive or IHC score 1+/FISH positive). All *P* values are two-sided, with *P* < 0.05 indicating statistical significance. *CI* confidence interval, *CY* peritoneal lavage cytology, *dM* distant metastasis excluding that detected in the peritoneum, by peritoneal lavage cytology, and in the liver, *H* hepatic metastasis, *N* lymph node metastasis, *P* peritoneal metastasis, *PS* performance status, *T* depth of tumor invasion (color figure online)

The incidence of low HER2 expression (IHC score 0/FISH positive or IHC score 1+/FISH positive) was 7.0 % (79 of 1136 patients); of these patients, 3.2 % of IHC score 0 cases (19 of 592 patients) and 11 % of IHC score 1+ cases (60 of 544 patients) were FISH positive (Table 3). In the univariate analysis, low HER2 expression was significantly correlated with sex, histological type, peritoneal metastasis, hepatic metastasis, depth of tumor invasion, and primary tumor location (Fig. 3a). Finally, multivariate logistic regression analysis revealed that age (65 years or older), intestinal type, and T1–T3 stage were independent factors related to low HER2 expression (Fig. 3b). We performed ad hoc analysis in the surgical specimen group. In the univariate analysis (*n* = 569), low HER2 expression was significantly correlated with sex (odds ratio 0.409, 95 % CI 0.178–0.940, *P* = 0.035), histological type (odds ratio 0.257, 95 % CI 0.131–0.507, *P* < 0.001), hepatic metastasis (odds ratio 4.598, 95 % CI 2.013–10.505, *P* < 0.001), depth of tumor invasion (odds ratio 0.405, 95 % CI 0.215–0.763, *P* = 0.005), and formalin concentration (odds ratio 1.949, 95 % CI 1.035–3.669, *P* = 0.039). However, multivariate logistic regression analysis revealed that there were no independent factors related to low HER2 expression in the 392 of 569 patients for whom data were available.

## Discussion

Previous studies reported that the rate of HER2 positivity (IHC score 3+ and/or FISH positive) in Japanese GC patients was approximately 10–20 % [5–7], but testing methods and interpretation criteria were not standardized. In this study, HER2 status was centrally assessed with a standardized method, which was used to prospectively interpret both the IHC data and the FISH data of the ToGA study; The rate of HER2 positivity was 21.2 % in Japanese patients, identical to the ToGA screening population [3]. The rate of HER2 positivity was reported as 27 % in Japanese patients in the ToGA study [4], higher than previously reported rates. This might be a result of bias toward patient selection from past reports [16–18], because the primary purpose of the ToGA study was to assess the clinical efficacy and safety of trastuzumab rather than to evaluate HER2 positivity. The incidence of higher HER2 protein expression (IHC score 2+/FISH positive or IHC score 3+; 15.6 %) and the proportions of FISH positivity in IHC score 0 and IHC score 1+ cases (3.2 and 11 %) were comparable with those reported in the ToGA study [3]. Similarly, the concordance between IHC and FISH in our results is consistent with that reported in the ToGA study.

A high correlation between HER2 positivity and histological subtype was reported by several authors [19–24]. In the ToGA study, HER2 positivity varied significantly according to histological subtype (intestinal type 31.8 %; diffuse type 6.1 %; mixed type 20 %) [3]; thus, intestinal type was strongly correlated with HER2 expression. Several reports indicated that intestinal type is associated with hematogenous metastasis, particularly to the liver [25], and with older age [26], whereas the diffuse type is adversely related to peritoneal dissemination [27]. In the present study, intestinal type, absence of peritoneal metastasis, and hepatic metastasis were shown to be independent factors related to HER2 positivity in a multivariate logistic regression analysis. This agrees with what is known about the histological type, i.e., intestinal or diffuse, and the association with accompanying hepatic or peritoneal metastasis, respectively.

Moreover, intestinal type, age (65 years or older), and T1–T3 stage were independent factors related to low HER2 expression (IHC score 0/1+ and FISH positive). This result reveals that HER2-related factors are associated with intestinal-type GCs. Diffuse-type GCs are more malignant than their intestinal-type counterparts, demonstrating early invasion into the muscularis propria [25]. A previous report demonstrated that diffuse-type advanced GC was significantly associated with advanced pathological T stage [28]. Thus, diffuse type is commoner in T4 tumors, whereas intestinal type is commoner in T1–T3 tumors. As intestinal type is the most robust factor related to HER2 expression, T1–T3 stage may be an independent factor related to low HER2 expression even in intestinal-type IHC score 0/1+ GC cases. However, the current study was limited by the extent and accuracy of the T staging, which was determined by either pathological or clinical diagnosis methods. To resolve these limitations, we performed ad hoc analyses for low HER2 expression (IHC score 0/FISH positive or IHC score 1+/FISH positive) in the surgical specimen group, because the T stage in the surgical samples was accurately determined pathologically. T1–T3 stage was statistically significantly correlated with low HER2 expression in the univariate analysis, but was not significantly correlated in the multivariate analysis. Likewise, intestinal type, sex, hepatic metastasis, and formalin concentration were statistically significantly associated with low HER2 expression in the univariate analysis; however, there were no significant differences in the multivariate analysis. The discrepancies in these analyses may result from the multivariate analysis being performed only in 392 of 569 cases owing to missing data in the remaining cases, thereby conferring a lack of statistical significance. Further studies are required to confirm this result, and considering these limitations, we cannot conclude that depth of tumor invasion is a factor related to low HER2 expression.

There are several factors that are reported to affect HER2 staining results, such as type of fixative, total fixation time, fixative pH, tissue type, and time before fixation. In the present study, we evaluated the relationship between HER2 expression and sampling conditions; however, the number of biopsy samples, formalin concentration, and formalin-fixation time had no significant effect on HER2 positivity and low HER2 expression. Unfortunately, the recommended conditions for fixation could not be adhered to in this study because the biopsy specimens and surgically resected specimens were mixed up and because correlations between formalin concentration and fixation time could not be undertaken. Moreover, the time before fixation (so-called cold ischemia) and the specimen size were unclear. Further prospective studies aiming to comprehensively evaluate the effects of formalin concentration, formalin-fixation time, and cold ischemia on HER2 testing are required.

There was concern that examination of gastric biopsy samples alone might introduce false-positive and/or false-negative data, because HER2 intratumoral heterogeneity in GC is observed in 20–70 % of HER2-positive tumors [13, 29] and is the major cause of discrepancies between biopsy samples and surgical specimens. In the multivariate analysis of the present study results, HER2-positivity rates in surgically resected tumors and biopsy samples were not significantly different, similar to the findings in the HER-EAGLE study [24]. However, these studies were limited in that the correlation between surgical specimens and biopsy samples was not paired, although this contrasts with the GERCOR study, where the overall concordance rate between surgical specimens and paired biopsy samples reached 94 % [12]. We also examined the concordance between predominant histological type and histological type with a HER2-positive component, which was determined as 81.3 % with the Lauren classification (data not shown). Approximately 20 % of cases showed a discrepancy; therefore, gastroenterologists should consider performing multiple biopsy sampling from varied collection sites to overcome tumor heterogeneity in GC.

In conclusion, HER2 expression in a Japanese GC population was similar in distribution to that identified in the ToGA study. Intestinal type was revealed as an independent factor related to both HER2 positivity and low HER2 expression.
